# Comparison of Baseline Characteristics, Sociodemographics, and Gynecological Risk Factors Associated with Secondary Infertility of Females in Pakistan

**DOI:** 10.1089/whr.2023.0145

**Published:** 2024-04-08

**Authors:** Wafa Fatima, Abdul Majeed Akhtar, Asif Hanif, Aima Gilani, Syed Muhammad Yousaf Farooq

**Affiliations:** ^1^ Faculty of Allied Health Sciences, University Institute of Public Health, The University of Lahore, Lahore, Pakistan.; ^2^ Trauma and Orthopedic Surgery, Salford Royal NHS Foundation Trust Hospital, Manchester, United Kingdom.; ^3^ Faculty of Allied Health Sciences, University Institute of Radiological Sciences and Medical Imaging Technology, The University of Lahore, Lahore, Pakistan.

**Keywords:** secondary infertility, risk factors, PCOS, PID, menorrhagia

## Abstract

**Introduction:**

Secondary infertility is characterized by the inability to conceive for a period of 1 year, after having previously conceived at least once.

**Objectives:**

To explore the risk factors of secondary infertility and compare sociodemographics and anthropometric variables of each studied group.

**Methods:**

Study was conducted at University Institute of Public Health, Faculty of Allied Health Sciences, The University of Lahore, collecting data from Gilani Ultrasound Center in 18 months after approval of synopsis. Total 690 females (345 cases and 345 controls) were enrolled. Participants were included in case group if they were 20–45 years of age, having any parity, and confirmed diagnosis of secondary infertility.

**Results:**

The mean age of cases and controls was 33.08 ± 4.17 years and 31.37 ± 4.36 years, respectively. The mean body mass index (BMI) in cases was 27.61 ± 4.27 kg/m^2^, and in controls the mean BMI was 25.52 ± 4.30 kg/m^2^. There was not a significant difference among religion that shows no association (*p* = 0.73) with secondary infertility as profession has association with it (*p* = 0.01). History of polycystic ovary syndrome, pelvic inflammatory disease, endometriosis, uterine fibroids, menorrhagia, intermenstrual bleeding, and history of abortion are associated with secondary infertility.

**Conclusions:**

While several sociodemographic features and medical disorders have been associated to secondary infertility, it is vital to stress that not all of these factors are controllable by medical therapy. Factors like age and certain medical issues may be unaffected by intervention. However, for controllable variables like BMI and certain medical diseases, focused therapies and lifestyle changes may reduce the chance of subsequent infertility.

## Introduction

Human infertility is a major health problem worldwide having its impact on the social, psychological, economical, and sexual life of a couple. The common definition of infertility is the inability of a couple to conceive following 12–24 months of unprotected intercourse. Infertility can be primary where a couple has never been able to conceive and secondary following a pregnancy.^1^ Approximately 10%–15% of couples suffer from infertility all over the world.^2^ Female factor is responsible in 35% and male factor in 45% of cases, while the rest of the couples either have combination of factors or unexplained infertility.^3,4^ Infertility is divided into primary and secondary infertility.^4^ It is recently reported that globally, infertility is a major health issue. Around 40 million couples actively sought treatment for infertility in 2010, of whom 34 million were in developing nations. There were ∼48.5 million infertile couples worldwide.^5^ The highest rate of infertility is said to be in females.^6^ Tubal factor infertility accounts for a large portion of female factor infertility. The most prevalent cause of tubal factor infertility is pelvic inflammatory disease (PID) and acute salpingitis. The incidence of tubal damage after one episode of pelvic infection is ∼12%, 23% after two episodes, and 54% after three episodes.^7^ However, the causes of infertility may be different in different geographic parts.^8^ An infertility evaluation is usually initiated after 1 year of regular unprotected intercourse in women under age 35 and after 6 months of unprotected intercourse in women age 35 years and older. However, the evaluation may be initiated sooner in women with irregular menstrual cycles or known risk factors for infertility, such as endometriosis, a history of PID, or reproductive tract malformations.^9^ Infertility in women can be diagnosed using various methods.^10^ Different risk factors are associated to secondary infertility like lifestyle related factors, such as obesity, diet, smoking, alcohol consumption, and chemical environments, and secondary factors related to human infertility such as unsafe methods of childbirth and postpartum period, as well as symptoms of sexually transmitted diseases.^11^ The other common factors responsible for infertility in females are anovulatory disorder, tubal factors, polycystic ovarian syndrome, peri-tubo-ovarian adhesions, endometriosis, uterine and cervical factors, *etc.*^12,13^ Traditional methods of statistical analysis are to determine a precise reason behind infertility and to provide effective predictors of treatment.^12^ The relationship between the analyzed factor and treatment outcome can be determined by univariate analysis. Secondary infertility in developing countries is mostly attributable to modifiable risk factors.^14^

There is a dearth of information on the prevalence and causes of secondary infertility from Pakistan. To date, literature is lacking on local population to explore the determinants of secondary infertility for comparison of multiple logistic regression and Artificial Neural Networks identification, and prediction of secondary infertility is limited in Pakistan and generally around the world. Therefore, this study is designed to explore sociodemographics and risk factors for secondary infertility in local population. After early determination of these risk factors through better modeling technique, we may highlight the issue to stakeholders and health care providers so that they could act for awareness of females and motivate them for modification of possible risk factors.

## Methods

**FIG. 1.**
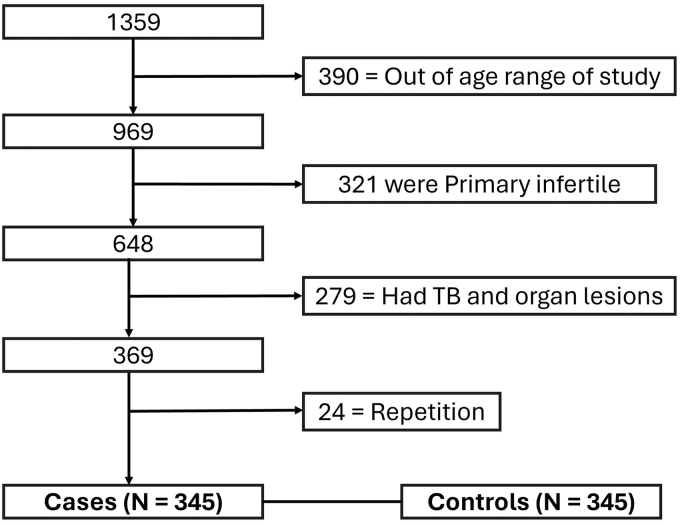
Flow chart for case identification.

A Case–control study was designed by nonprobability consecutive sampling. This study was conducted at University Institute of Public health by taking data from Gilani Ultrasound Center during time of 18 months after approval of synopsis. This study was approved by the Ethics Committee of Gilani Ultrasound Center and The University of Lahore also. A total of 690 females (345 cases and 345 controls) were taken. Inclusion criteria for cases were females with age of 20–45 years, having any parity, and diagnosed as secondary infertility (as per operational definition). Inclusion criteria for control group were females not diagnosed with secondary infertility, couples who were living together for at least 12 months, couples with male factor infertility, and with age of 20–45 years. Exclusion criteria for cases and controls were couples who had not lived together for at least 12 months, couples with male factor infertility, and infertile female with H/O Tuberculosis. The rules and regulations set by the Ethics Committee of University of Lahore were followed while conducting the research, and the rights of the research participants were respected. Written informed consent (attached) was taken from all the participants. A STROBE flow diagram represents how many patients were excluded from study on exclusion criteria (Fig. 1). All information and data collection were kept confidential. Demographic information, that is, age, years since marriage, and duration of infertility, was collected. Complete history and examination were done and recorded. All information was recorded on question answer sheet and analyzed.

All data were entered and analyzed using Stata program. In descriptive analysis, for quantitative data mean ± standard deviation and median ± interquartile range were used. Independent sample *t*-test (where data were normal) and Mann–Whitney *U* test were applied (where data were not normal). For categorical data frequency (%) was used and Chi-square test was applied to see association between cases and controls and other factors. *p*-Value ≤0.05 was considered as significant.

## Results

The mean age of cases (females with secondary infertility) and controls was 33.08 ± 4.17 years and 31.37 ± 4.36 years, respectively. The median age was statistically higher in cases (34.0 ± 6 years) than control (30.0 ± 7 years), *p*-value <0.05. The mean age at marriage of cases was 24.33 ± 4.76 years and of control was 24.59 ± 2.96 years. The median age at marriage was statistically insignificant in cases and controls, that is, 25.0 ± 6 years and 25.0 ± 5 years, *p*-value >0.05. The mean body mass index (BMI) in cases was 27.61 ± 4.27 kg/m^2^ and in controls the mean BMI was 25.52 ± 4.30 kg/m^2^. The median BMI was statistically higher in cases than controls, that is, 27.64 ± 7.56 than 24.77 ± 6.73, *p*-value <0.05. The mean education of females in cases and control groups was 11.61 ± 5.72 years and 11.47 ± 5.43 years. The median education was statistically insignificant in cases and controls, that is, 14.0 ± 6 and 12.0 ± 5, respectively, *p*-value >0.05. The average monthly income was 64.05 ± 84.20 in cases and was 72.52 ± 106.85 in control group. The median monthly income was statistically same in cases and control groups, that is, 45.0 ± 30 and 32.0 ± 50, *p*-value >0.05 **(**Table 1**)**.

**Table 1.** Comparison of Baseline Characteristics of Participants

	Median ± IQR	***z***-test^a^	***p***
Age (years)			
Cases	34.0 ± 6	−5.20	<0.001^b^
Controls	30.0 ± 7		
Total	32.0 ± 6		
Age at marriage (years)			
Cases	25.0 ± 6	−2.98	>0.05
Controls	25.0 ± 5		
Total	25.0 ± 6		
BMI			
Cases	27.64 ± 7.56	−5.60	<0.001^b^
Controls	24.77 ± 6.73		
Total	25.86 ± 7.01		
Education (years)			
Cases	14.0 ± 6	−1.09	0.273
Controls	12.0 ± 5		
Total	13.0 ± 6		
Monthly income ^c^1000 (Rs.)			
Cases	45.0 ± 30	−1.06	0.289
Controls	32.0 ± 50		
Total	45.0 ± 43		

^a^ Results by the *z* test were considered statistically significant if *Z* test score is below −1.960 or above +1.960.

^b^ Highly significant.

^c^ Significant; Mann–Whitney *U* test was applied.

BMI, body mass index; IQR, interquartile range.

In case group there were 326 (94.5%) Muslim females and 19 (5.5%) were Christian, while in controls there were 328 (95.1%) Muslim and 17 (4.9%) were Christian females; there was no association between infertility and religion, *p*-value >0.05. There were 128 (37.1%) working females and 217 (62.9%) were housewives in cases, while in control groups there were 97 (28.1%) working and 248 (71.9%) were housewives. The working females were statistically higher in cases than controls, *p*-value <0.05. Among cases, 59 (17.1%) females belonged to lower, 265 (76.8%) were from middle, and 21 (6.1%) females were from upper class, while in controls group, there were 63 (18.3%) females who were from upper, 267 (77.4%) were from middle, and 15 (4.3%) females were from lower class. There was no association between study groups and socioeconomic class, *p*-value >0.05 (Table 2).

**Table 2.** Comparison of Sociodemographic Variables in Both Study Groups

	Groups		***χ*** ^2^	***p***	OR (95% CI)
	Cases	Controls			
Religion					
Islam	326 (94.5%)	328 (95.1%)	0.12	0.73	0.89 (0.45–1.74)
Christian	19 (5.5%)	17 (4.9%)			
Profession					
Working	128 (37.1%)	97 (28.1%)	6.34	0.01^*^	1.51 (1.09–2.08)
Housewife	217 (62.9%)	248 (71.9%)			
Socioeconomic status					
Lower	59 (17.1%)	63 (18.3%)	1.14	0.57	—
Middle	265 (76.8%)	267 (77.4%)			
Upper	21 (6.1%)	15 (4.3%)			

CI, confidence interval; OR, odds ratio.

**FIG. 2.**
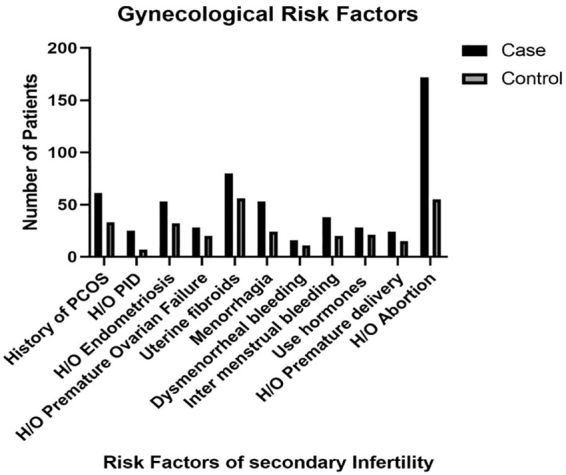
Gynecological risk factors associated with secondary infertility.

There was a significant association of studied group with respect to history of polycystic ovary (17.7% vs. 9.6%), PID (7.2% vs. 2.0%), history of endometriosis (15.4% vs. 9.3%), uterine fibroids (23.2% vs. 16.2%), history of menorrhagia (15.4% vs. 7.2%), intermenstrual bleeding (11% vs. 5.8%), and history of abortion (both miscarriage/spontaneous abortion and medical/therapeutic abortion) (49.9% vs. 15.9%). The risk of secondary infertility was higher for females having history of polycystic ovary syndrome (PCOS), that is, odds ratio (OR) = 2.03 (1.29–3.19), for history of PID, that is, OR = 3.77 (1.61–8.84), for history of endometriosis, that is, OR = 1.78 (1.11–2.83). There were 1.56 times higher chances for secondary infertility in females having uterine fibroids, that is, OR = 1.56 ((1.07 2.28), for menorrhagia, that is, OR = 2.32 (1.41–3.84), for intermenstrual bleeding, that is, OR = 2.01 (1.15–3.53), and for history of abortion, that is, OR = 5.24 (3.67–7.49) (Table 3 and Fig. 2).

**Table 3.** Comparison of Gynecological History in Both Study Groups

	Groups		***χ*** ^2^	***p***	OR (95% CI)
	Cases	Controls			
History of PCOS					
Yes	61 (17.7%)	33 (9.6%)	9.66	0.002^*^	2.03 (1.29–3.19)
No	284 (82.3%)	312 (90.4%)			
History of PID					
Yes	25 (7.2%)	7 (2.0%)	10.62	0.001^*^	3.77 (1.61–8.84)
No	320 (92.8%)	338 (98.0%)			
History of endometriosis					
Yes	53 (15.4%)	32 (9.3%)	5.92	0.02^*^	1.78 (1.11–2.83)
No	292 (84.6%)	313 (90.7%)			
History of premature ovarian failure					
Yes	28 (8.1%)	20 (5.8%)	1.43	0.23	1.44 (0.74–0.79)
No	317 (91.9%)	325 (94.2%)			
Uterine fibroids					
Yes	80 (23.2%)	56 (16.2%)	5.28	0.02^*^	1.56 (1.07–2.28)
No	265 (76.8%)	289 (83.8%)			
Menstrual regularity					
Regular	230 (66.7%)	234 (67.8%)	0.11	0.75	0.95 (0.69–1.30)
Irregular	115 (33.3%)	111 (32.2%)			
Menorrhagia					
Yes	53 (15.4%)	25 (7.2%)	11.33	0.001^*^	2.32 (1.41–3.84)
No	292 (84.6%)	320 (92.8%)			
Dysmenorrheal bleeding					
Yes	16 (4.6%)	11 (3.2%)	0.96	0.33	1.48 (0.68–3.23)
No	329 (95.4%)	334 (96.8%)			
Intermenstrual bleeding					
Yes	38 (11%)	20 (5.8%)	6.1	0.01^**^	2.01 (1.15–3.53)
No	307 (89%)	325 (94.2%)			
Used hormones to regulate menstruation					
Yes	28 (8.1%)	21 (6.1%)	1.08	0.3	1.36 (0.76–2.45)
No	317 (91.9%)	324 (93.9%)			
H/o premature delivery					
Yes	24 (7%)	15 (4.3%)	2.2	0.14	1.65 (0.85–3.19)
No	321 (93%)	330 (95.7%)			
History of abortion					
Yes	172 (49.9%)	55 (15.9%)	89.89	<0.001^**^	5.24 (3.67–7.49)

PCOS, polycystic ovary syndrome; PID, pelvic inflammatory disease.

The secondary infertility was also not associated with previous birth history, that is, skilled birth (95.9% and 96.2%), attendant washing hands before delivery (96.5% and 97.7%), clean sheets used before delivery (93.3% and 95.1%), clean instruments used before delivery (93.6% and 92.8%), gloves used before delivery (94.2% and 95.4%), and use of intravaginal preparation (95.7% and 97.1%), *p*-value >0.05 (Table 4).

**Table 4.** Comparison of Previous Birth History in Both Groups

	Groups		***χ*** ^2^	***p***	OR (95% CI)
	Cases	Controls			
Skilled birth attendant					
Yes	331 (95.9%)	332 (96.2%)	0.04	0.84	0.93 (0.43–2.0)
No	14 (4.1%)	13 (3.8%)			
Washing hands before delivery					
Yes	333 (96.5%)	337 (97.7%)	0.82	0.36	0.66 (0.27–1.63)
No	12 (3.5%)	8 (2.3%)			
Clean sheets used before delivery					
Yes	322 (93.3%)	328 (95.1%)	0.96	0.33	0.73 (0.38–1.38)
No	23 (6.7%)	17 (4.9%)			
Clean instruments used before delivery					
Yes	323 (93.6%)	320 (92.8%)	0.21	0.65	1.15 (0.63–2.08)
No	22 (6.4%)	25 (7.2%)			
Gloves used before delivery					
Yes	325 (94.2%)	329 (95.4%)	0.47	0.49	0.79 (0.40–1.55)
No	20 (5.8%)	16 (4.6%)			
Use of intravaginal preparation					
Yes	330 (95.7%)	335 (97.1%)	1.04	0.31	0.67 (0.29–1.48)
No	15 (4.3%)	10 (2.9%)			

## Discussion

After 12–24 months of consistent unprotected intercourse, couples who are unable to conceive are said to be infertile. Infertility varies by individuals. In both men and women, the fertility process is complex. Around 10%–15% of all couples experience infertility.^15^ The single most significant factor affecting both spontaneous and treatment-related conception is female age. The threshold for advanced reproductive age lacks a universally agreed-upon definition; however, it is generally acknowledged that 35 years marks a significant point in terms of fertility.^16^ In this study patients presenting with secondary infertility were of age, that is, 33.08 ± 4.17 years. A study was conducted in 2010 by Nosheen,^17^ reported mean age in secondary infertility was 32, while Talib reported 29.4 years of mean age in secondary infertility.^18^ The healthy development of female reproductive functions depends on adipose tissue. The numerous reproductive issues seen in infertile women are caused by an association between fat and hyperinsulinemia, hyperandrogenism, and abnormal hormone production, including leptin.^19,20^ Factors related to nutrition and lifestyle that impact fertility encompass conditions such as anemia, weight imbalances, and smoking. The American Society for Reproductive Medicine emphasizes that 12% of infertility instances arise from being either underweight or overweight.^21^ According to current study, women with obesity had 1.45 times higher chances of secondary infertility. A study found BMI significantly higher in females with secondary infertility.^22^ Yet, research in China has indicated that the highest incidence of infertility in women is observed among those who are underweight.^23^ In this study females with PID had higher chances of secondary infertility, that is, 3.77 times, same results have been reported by a study in Peshawar^24^ followed by PCOS, that is, 2.03 times, endometriosis, that is, 1.78 times, and uterine fibroids, that is, 1.56 times. By comparing our results with other studies, PCOS, endometriosis, and uterine fibroids are also found to be the cause of secondary infertility.^17,23^ Sultana and her team documented parallel results in a local study, noting occurrences of ovulation failure in 60% of cases, polycystic ovarian disease in 32%, bilateral tubal occlusion in 8%, and pelvic adhesions in 24%.^25^ Another interesting finding in our study was the significant association of abortion with secondary infertility with 5.24 times more chances. A study was conducted in Nigeria in which induced abortion and postabortion sepsis were the most important risk factors for secondary infertility.^26^ Another study found association between abortion and secondary infertility.^27^

Our study highlights several significant factors associated with secondary infertility. Female age, particularly after 35 years, emerges as a crucial determinant of fertility. Lifestyle-related factors, such as obesity and diabetes, also play a notable role. In addition, conditions like polycystic ovarian syndrome, endometriosis, and uterine fibroids contribute to secondary infertility. Moreover, tubal disease, often linked to PID, stands out as a major cause of secondary infertility. Importantly, abortion related complications were found to significantly increase the risk of secondary infertility.

Several limitations should be considered when analyzing this secondary infertility research. The small age range of participants may limit the findings' applicability. The cross-sectional research design makes causal linkages and temporal dynamics between risk variables and secondary infertility difficult to establish. Recall bias may affect lifestyle characteristics and medical histories indicated by self-reported data. Finally, a single-center research may not reflect different health care contexts and procedures. Addressing these limitations in future research may strengthen and apply the study's findings.

## Conclusion

The purpose of this study was to identify social and demographic factors among females in both case and control groups, such as age, BMI, profession, monthly income, and medical histories such as PCOS, PID, endometriosis, uterine fibroids, menorrhagia, intermenstrual bleeding, and abortion history. While some sociodemographic characteristics and medical illnesses have been linked to secondary infertility, it is important to highlight that not all of these factors are changeable *via* medical treatment. Age and certain medical issues may be unaffected by intervention. However, for controllable variables like BMI and certain medical diseases, focused therapies and lifestyle changes may reduce the chance of subsequent infertility. It is critical to grasp the subtle nature of these linkages and to evaluate the constraints of trying to adjust nonmodifiable elements.

## Abbreviations Used

BMI: body mass index
CI: confidence interval
IQR: interquartile range
PCOS: polycystic ovary syndrome
PID: pelvic inflammatory disease
PCOD: polycystic ovarian disease
OR: odds ratio
